# COPD in Brazil: where there’s smoke…

**DOI:** 10.36416/1806-3756/e20250235

**Published:** 2025-07-22

**Authors:** Frederico Leon Arrabal Fernandes

**Affiliations:** 1. Divisão de Pneumologia, Instituto do Coração, Hospital das Clínicas, Faculdade de Medicina, Universidade de São Paulo, São Paulo (SP) Brasil.

Brazil is a developing country. As such, its low-income population is more vulnerable to environmental exposures and the harms associated with inhaled pollutants, especially cigarette smoke and biomass burning. Several studies have shown that individuals from lower socioeconomic classes have a higher prevalence of smoking and are more frequently exposed to the burning of wood, charcoal, or other biomass components for heating, energy, and cooking.^1^ ^,^ ^2^ This remains a common practice in rural areas and urban outskirts.^1^ ^,^ ^2^

This environmental vulnerability is compounded by limited access to health care services, especially in rural and remote regions, where primary care infrastructure may be weak and access to complementary tests-particularly spirometry-may be virtually nonexistent. Thus, populations at a higher risk of developing chronic respiratory diseases such as COPD paradoxically have less access to the resources necessary for their diagnosis and treatment. Reducing this inequality requires public policies focused on expanding health care access, strengthening primary health care (PHC), increasing availability of diagnostic tools, and implementing educational initiatives that consider the social, cultural, and environmental realities of these communities.^3^

The multicenter study “Sociodemographic and clinical characteristics of individuals exposed to smoking or biomass smoke and followed at primary health care centers in Brazil: a multicenter study,”^4^ published in the present issue of the *Jornal Brasileiro de Pneumologia*, addresses a highly relevant topic for PHC: the profile of individuals exposed to risk factors for respiratory diseases, such as tobacco smoke and biomass smoke. Conducted at four primary health care units in southern and southeastern Brazil, the study evaluated 737 patients aged 35 years or older, including smokers, former smokers, and individuals exposed to biomass.

The results revealed a population predominantly composed of women (56.3%), with a mean age of 57.7 years, low educational level (over half with nine or fewer years of schooling), and belonging to lower socioeconomic classes (C2/D/E). A noteworthy finding was the high prevalence of overweight and obese individuals (71.5%), along with common comorbidities such as hypertension (51.3%), depression (27.4%), and diabetes (24.3%).^4^

Regarding respiratory symptoms, high rates of cough (37.3%), wheezing (33.8%), and sputum production (27.4%) stand out. Although most patients reported only mild dyspnea (75.1% with mMRC 0-1), the majority had a COPD Assessment Test (CAT) score above 10 points, characterizing them as highly symptomatic. Biomass exposure, though less prevalent, was significantly associated with a greater impact of respiratory symptoms on CAT scores.^4^

Statistical analyses showed that male sex and older age were associated with higher levels of dyspnea, whereas higher BMI and lower socioeconomic status were linked to worse CAT scores. Interestingly, no statistically significant association was found between smoking or obesity and the most prevalent comorbidities, raising hypotheses about limitations inherent to cross-sectional study designs and reinforcing the need for longitudinal research.^4^

The article highlights the strategic role of PHC in addressing vulnerable populations and in the early detection of chronic respiratory diseases.^4^ The significant presence of symptoms even in early stages and the frequency of comorbidities point to a critical opportunity for preventive actions and health education at the primary care level. It is also important to emphasize the negative effects of biomass exposure, often overlooked in clinical practice and public policies, but with measurable impact on the quality of life of patients.

In summary, this is an important contribution that sheds light on the clinical and sociodemographic profile of a high-risk population often rendered invisible in large respiratory disease studies. The findings underscore the potential of PHC as a strategic space for prevention, screening, and health education and point to the urgent need for integrated public policies that consider the social and environmental contexts of patients.

One particularly striking finding in the study was the high frequency of respiratory symptoms among participants. Cough, wheezing, and sputum production were reported by more than a third of the sample, in stark contrast to the low prevalence of chronic respiratory disease diagnoses, such as COPD, recorded in the PHC units. This discrepancy is notable when compared with other cross-sectional population studies such as the so-called PLATINO study,^5^ which estimated a COPD prevalence of 15.8% in Brazil among adults aged 40 or older, but only 6.8% in the current study.^4^ The gap suggests a worrisome underdiagnosis of COPD in PHC, possibly linked to the low awareness of symptoms or the normalization of respiratory illness in vulnerable populations, perhaps because of the lack of routine spirometry.

It is worth emphasizing that the epidemiological and anthropometric profile of the sample, characterized by having advanced age, being overweight, having a low socioeconomic status, and being highly exposed to risk factors, such as smoking and biomass smoke, is highly consistent with the typical profile of COPD patients; and yet, the prevalence of this disease, which is the third leading cause of death globally, was relatively low. These findings reinforce the importance of strengthening PHC to actively screen for respiratory diseases through systematic use of spirometry, professional training, and integration with specialized care networks for proper diagnosis and management.

The discrepancy between the high prevalence of respiratory symptoms and the low rate of chronic respiratory disease diagnoses, such as COPD, exposes a critical gap in the diagnostic approach of PHC. This finding underscores the urgent need to enhance the capacity of PHC teams for active case finding of patients with COPD and other chronic lung conditions. Early detection of these diseases relies not only on clinical recognition of symptoms and risk factors but also on the use of objective diagnostic tools, especially spirometry.

Despite being recommended by both national and international guidelines as a first-line diagnostic tool for COPD, spirometry remains underutilized in Brazilian PHC due to structural barriers, lack of technical training, and weak integration with specialized services. Making spirometry more accessible, widely available, and easier to request is essential to enable timely diagnosis and reduce the underdiagnosis so well documented in this^4^ and other studies.

COPD diagnosis remains precarious in the PHC setting, even among populations with well-established risk factors. Much of the problem stems from the disease’s insidious onset, with symptoms emerging slowly and progressively, making them harder for patients to recognize. Dyspnea on exertion is often attributed to aging or a sedentary lifestyle, while chronic cough and sputum are considered “normal” among smokers. As a result, many individuals do not seek health care facilities for these symptoms and, even when they do for other reasons, tend not to report them spontaneously.

Patients with known risk factors, such as smokers and former smokers, individuals with a history of pulmonary tuberculosis, those exposed to biomass smoke, or with a childhood history of recurrent respiratory infections, should be systematically and thoroughly assessed, spirometry being proactively offered even in the absence of prominent respiratory complaints. The fact that spirometry is mostly restricted to large centers and tertiary care facilities is a significant barrier to more proactive and preventive approaches.

Relying solely on spontaneous demand is insufficient. The implementation of active screening strategies, along with ongoing health professional training and a robust diagnostic support network, can transform PHC into an effective setting for early COPD interception with direct benefits for morbidity, quality of life, and health care costs.

Active case finding, expanded access to spirometry, professional training, and stronger integration across levels of care must be central to public health strategies for tackling COPD. Promoting early diagnosis is not merely a technical goal, it is a matter of social justice, particularly for vulnerable populations who silently face risk factors and develop disabling, yet preventable, symptoms.

We live within a perverse logic: those who are the most susceptible are also those who are the most neglected, which leads to chronic underdiagnosis of COPD especially in its early stages, precisely when interventions could change the trajectory of the disease. Therefore, PHC teams must adopt a proactive stance in investigating respiratory symptoms, identifying risk factors, and adopting clear strategies for timely screening. The popular saying “where there’s smoke, there’s fire” aptly applies to COPD: in the presence of any significant exposure to tobacco, biomass, or other risk factors, one must consider the possibility of lung disease even when patients do not spontaneously report symptoms.
